# Characterization of a Thermostable Endolysin of the *Aeribacillus* Phage AeriP45 as a Potential *Staphylococcus* Biofilm-Removing Agent

**DOI:** 10.3390/v16010093

**Published:** 2024-01-07

**Authors:** Natalia N. Golosova, Yana A. Khlusevich, Vera V. Morozova, Andrey L. Matveev, Yulia N. Kozlova, Artem Y. Tikunov, Elizaveta A. Panina, Nina V. Tikunova

**Affiliations:** Institute of Chemical Biology and Fundamental Medicine, Siberian Branch of the Russian Academy of Sciences, 630090 Novosibirsk, Russia; n.golosova@g.nsu.ru (N.N.G.); morozova@niboch.nsc.ru (V.V.M.); guterus@gmail.com (A.L.M.); ulona79@mail.ru (Y.N.K.); arttik1986@gmail.com (A.Y.T.); e.panina@g.nsu.ru (E.A.P.)

**Keywords:** thermostable endolysin, cell-binding domain CBD, phage, *Staphylococcus aureus*, *Aeribacillus pallidus*, antibiotic resistance, antimicrobial agent, biofilm

## Abstract

Multidrug-resistant Gram-positive bacteria, including bacteria from the genus *Staphylococcus*, are currently a challenge for medicine. Therefore, the development of new antimicrobials is required. Promising candidates for new antistaphylococcal drugs are phage endolysins, including endolysins from thermophilic phages against other Gram-positive bacteria. In this study, the recombinant endolysin LysAP45 from the thermophilic *Aeribacillus* phage AP45 was obtained and characterized. The recombinant endolysin LysAP45 was produced in *Escherichia coli* M15 cells. It was shown that LysAP45 is able to hydrolyze staphylococcal peptidoglycans from five species and eleven strains. Thermostability tests showed that LysAP45 retained its hydrolytic activity after incubation at 80 °C for at least 30 min. The enzymatically active domain of the recombinant endolysin LysAP45 completely disrupted biofilms formed by multidrug-resistant *S. aureus*, *S. haemolyticus*, and *S. epidermidis*. The results suggested that LysAP45 is a novel thermostable antimicrobial agent capable of destroying biofilms formed by various species of multidrug-resistant *Staphylococcus*. An unusual putative cell-binding domain was found at the C-terminus of LysAP45. No domains with similar sequences were found among the described endolysins.

## 1. Introduction

For many years, antibiotics were considered highly effective agents for the therapy of staphylococcal infections, although resistant bacteria soon began to be identified. In the 1960s, methicillin-resistant hospital strains of *Staphylococcus aureus* (MRSA) were discovered [1]. Then, MRSA strains spread rapidly around the world and today pose a serious problem. The emergence of strains with multiple antibiotic resistance is of primary concern [2]. In 2017, the World Health Organization included *S. aureus* in the list of the most dangerous agents resistant to antibiotics.

Many *Staphylococcus* species are commensals of human and animal skin and mucosal surfaces. However, they could cause opportunistic infections in humans. The main human pathogens are *S. aureus*, *Staphylococcus epidermidis*, and *Staphylococcus haemolyticus*. They can cause a variety of diseases from minor skin infections to life-threatening ones such as pneumonia, meningitis, and sepsis [3]. The most pathogenic member of the genus is coagulase-positive *S. aureus*. Its high pathogenicity is associated with the production of a wide range of virulence factors, namely, protein A, coagulase, collagenase, hyaluronidase, hemolysins, lipases, various toxins, adhesive proteins, and proteins that promote biofilm formation [3]. Infections caused by *S. aureus* lead to tens of thousands of fatal cases annually [3]. *S. epidermidis* is the most pathogenic member among the coagulase-negative staphylocococci, which also include *S. haemolyticus, Staphylococcus saprophyticus, Staphylococcus warneri*, and some other species. Infections caused by *S. epidermidis* are characterized by a chronic course, with biofilm formation that increases resistance to antibiotics. *S. epiderm* idis is a frequent cause of opportunistic infections, namely, implant-associated infections, prosthetic valve endocarditis, and pacemaker infections [4,5,6]. *S. epidermidis* is also responsible for 30–40% of nosocomial bloodstream infections, which are caused by biofilm-associated catheter infections [5]. Bloodstream infections are difficult to treat and can lead to serious complications including sepsis, septic shock, and infective endocarditis [6]. During infection, *S. epidermidis* produces phenol-soluble modulins that negatively affect human erythrocytes and leukocytes [7,8,9]. It was recently shown that the proportion of strains with multiple antibiotic resistance may be significantly higher among *S. epidermidis* than among *S. aureus* [10], which makes *S. epidermidis* a serious pathogen. So, the development of new effective antimicrobials is required.

Endolysins of phages infecting Gram-positive bacteria are one of the promising alternatives to antibiotics [11]. These phage proteins have catalytic activity that disrupts bonds in peptidoglycans in the bacterial cell wall. The cell wall of staphylococci consists of a multilayer macromolecular network of peptidoglycan, teichoic acids, and surface proteins. Glycan strands are cross-linked by short peptides attached to each *N*-acetylmuramic acid (MurNAc) residue [12,13,14,15].

Endolysins are required at the late stages of the life cycle of bacteriophages for the release of viral particles [11]. Endolysins are predominantly hydrolases [11,12], and phage endolysins are divided into three main groups: amidase, glucosidase, and peptidase [12]. Phage endolysins that hydrolyze the peptidoglycan of Gram-negative and Gram-positive bacteria differ due to differences in the structure of the host bacterial cell wall. Endolysins of phages that infect Gram-positive bacteria usually consist of two functional domains: the cell-binding domain (CBD) and the enzymatically active domain (EAD). In contrast, endolysins of phages of Gram-negative bacteria are small molecules and mainly consist of a single EAD [16]. The use of endolysins as antimicrobial agents has significant advantages over the use of antibiotics, such as low toxicity to mammalian cells, specificity to the target bacterium, and minimized potential for the development of resistance [14,15,16,17,18,19,20].

Phage AP45, isolated from a soil sample collected in the Geyser Valley, Kamchatka Peninsula, Russia, shows specificity to the thermophilic bacterium *Aeribacillus pallidus* CEMTC 656, also isolated from the Geyser Valley [21]. Recombinant endolysin LysAP45 was constructed using the appropriate gene from the AP45 genome. This endolysin showed the ability to hydrolyze peptidoglycans from the cell wall of a sensitive *A. pallidus* strain, which was shown by zymography [21]. As LysAP45 was produced mainly as an insoluble protein and its properties have not been sufficiently studied, a new producer strain was constructed, and the hydrolytic activity of purified LysAP45 against staphylococcal biofilms was tested. In addition, the thermal stability of LysAP45 was examined.

## 2. Materials and Methods

### 2.1. Bacterial Strains and Growth Conditions

The bacterial strains *Escherichia coli E. coli* DH5alphaF`, *E. coli* M15, *A. pallidus* CEMTC 656, *S. aureus* CEMTC 675, *S. aureus* 1685, *S. aureus* 1733, *S. epidermidis* strains 2043, *S. epidermidis* CEMTC 2079, *S. haemolyticus* CEMTC 3413, *S. haemolyticus* CEMTC 3753, *S. warneri* CEMTC 2062, *S. warneri* CEMTC 4154, *S. saprophyticus* CEMTC 3872, and *S. saprophyticus* CEMTC 6829 were obtained from the Collection of Extremophile Microorganisms and Type Cultures (CEMTC) of ICBFM SB RAS.

The taxonomy of *Staphylococcus* spp. was confirmed by sequencing the 16S rRNA gene. All used staphylococcal strains were isolated from clinical specimens previously [7], and their antibiotic resistance was assessed by the Oxoid disk diffusion method in accordance with the EUCAST recommendations; the results are indicated in Table S1. These strains were used for the assessment of the antibacterial activity of the endolysin LysAP45. The *E. coli* strains DH5alpha F` and M15 were used for the production of the recombinant endolysin LysAP45. The *A. pallidus* strain CEMTC 656 was grown in nutrient broth (NB, Condalab, Madrid, Spain). Other bacterial strains were grown in Luria Bertani (LB) medium/agar (1.5% *w*/*v*) (10 g/L peptone, 5 g/L yeast extract, 10 g/L NaCl, pH: 7.0, Becton Dickinson, Franklin Lakes, NJ, USA). The planktonic bacterial cells were cultivated in an orbital shaker incubator with 150 rpm at 37 °C (Infors HT, Bottmingen, Switzerland).

### 2.2. Phage AP45 Propagation and Host Range Experiments

Phage AP45 propagation was described previously [21]. The host range for the AP45 phage was tested by spotting serial phage dilutions onto freshly prepared lawns of the above-mentioned *Staphylococcus* strains, as described previously [22]. Spotting of the same dilutions of AP45 onto *A. pallidus* CEMTC 656 was used as a positive control.

### 2.3. In Silico Structural Analysis of Putative Endolysins

The phage endolysin LysAP45 (protein id: APC46516.1) is encoded by ORF 65 (47766–48632 b.p.) and consists of 288 amino acid residues (aa). The domains of the endolysin LysAP45 were identified using the protein database from NCBI (https://www.ncbi.nlm.nih.gov/protein, accessed on 21 September 2022) and InterProScan software (https://www.ebi.ac.uk/interpro, accessed on 20 September 2022) [23]. The three-dimensional (3D) model of LysAp45 was predicted using AlphaFold2 available through the online service (https://colab.research.google.com/github/sokrypton/ColabFold/blob/main/AlphaFold2.ipynb, accessed on 21 August 2023) [24]. Ribbon and surface representations of LysAp45 were visualized using the UCSF chimera molecular visualizer, version 1.15 [25]. The protein solubility of LysAP45 was calculated using the SoluProt [26] and Protein-sol [27] on-line services (https://protein-sol.manchester.ac.uk accessed on 10 September 2023, https://loschmidt.chemi.muni.cz/soluprot, accessed on 10 September 2023). The ligand-binding site was predicted using the meta-server approach to protein–ligand-binding site prediction COFACTOR [28].

### 2.4. Production and Purification of the Recombinant Endolysin LysAP45

The plasmid DNA pQE-70/LysAP45 encoding the recombinant endolysin LysAP45 was constructed previously (Figure S1) [21]. To produce the recombinant endolysin, *E. coli* M15 or *E. coli* DH5alpha F` cells were transformed with the pQE-70/LysAP45 plasmid and incubated overnight in a dish with LB/agar, containing ampicillin (50 μg/mL). Then, *E. coli* M15-pQE-70/LysAP45 cells were grown to OD_600_ = 0.6–0.8, in LB medium with ampicillin (50 μg/mL). The production of the recombinant endolysin was induced by adding isopropyl β-D-1-thiogalactropyranoside (IPTG, Sigma Aldrich, St. Louis, MO, USA) at a concentration of 100 µM. The induced cells were cultivated at 25 °C with shaking (150 rpm), overnight. The next day, the cells were centrifuged 10 min at 6000× *g* and resuspended in 50 mM Tris-HCl pH 8.0. The cell suspension was lysed by sonication (34% power) for 10 minutes on ice, using the ultrasonic Sonopuls HD 2070 homogenizer (Bandelin, Berlin, Germany). The expression level of the recombinant protein and its cell localization were analyzed using 12.5% SDS-PAGE and the Gel Doc XR+ gel documentation system (Biorad, Hercules, CA, USA).

The recombinant endolysin LysAP45 was purified using metal chelate chromatography on Ni-NTA agarose (Qiagen, Venlo, The Netherlands) from the soluble cytoplasm fraction according to the manufacturer’s protocol. Briefly, a chromatography column containing Ni-NTA agarose (Sigma Aldrich, St. Louis, MO, USA) was equilibrated with buffer A (50 mM NaH_2_PO_4_, pH 8.0, 300 mM NaCl, 5 mM Tris-HCl), and the cytoplasmic fraction of *E. coli* M15-pQE-70/LysAP45 cells was flowed through the column. Then, the column was washed with buffer A containing 25 mM imidazole to remove nonspecifically bound proteins. Buffer A, containing 100 mM imidazole, was used to elute the recombinant endolysin LysAP45. In an eluate that contained the recombinant endolysin LysAP45, the buffer was replaced with a storage buffer (50 mM Tris-HCl pH 7.5, 300 mM NaCl) by dialysis. Protein concentration was measured using the Qubit protein assay kit (Thermo Fisher Scientific, Waltham, MA, USA) on a Qubit 4 Fluorometer (Thermo Fisher Scientific, Waltham, MA, USA).

### 2.5. Zymography Using Peptidoglycans of S. aureus, S. haemolyticus, S. epidermidis, S. warneri, and S. saprophyticus

Peptidoglycans from staphylococcal cell walls were isolated according to aa previously described method [29]. Briefly, *Staphylococcus* spp. cells were grown to OD_600_ = 1–1.5 in 1 L of LB medium at 37 °C, at 150 rpm. The cells were pelleted by centrifugation for 10 min at 10,000× *g*, resuspended in 8 mL of 4 M LiCl, and boiled in a water bath for 15 min, followed by centrifugation. The precipitate was resuspended in deionized water and sonicated (40% power) for 30 min on ice, using the Sonopuls HD 2070 homogenizer (Bandelin, Berlin, Germany). The suspension was centrifuged at 11,000× *g* for 10 min; the precipitate was resuspended in 10 mL of 4% SDS, boiled in a water bath for 15 min, and centrifuged for 10 min at 11,000× *g* at room temperature. The resulting precipitate was resuspended in 10 mL of 1 M NaCl and centrifuged again (10 min, 11,000× *g*); this procedure was repeated until the precipitate became colorless. The obtained precipitate was suspended in 2–3 mL of deionized water and centrifuged for 10 min at 11,000× *g*. The washing step was repeated 1–2 times. Finally, the precipitate was resuspended in 2 mL of deionized water, containing 0.02% sodium azide, and stored at 4 °C. The peptidoglycan concentration was measured using a spectrophotometer. OD_540_ = 1.0 approximately corresponded to the peptidoglycan concentration of 1 mg/mL.

The recombinant LysAP45 was analyzed by SDS-PAGE in the presence of 0.1 mg/mL of peptidoglycan from the staphylococcal cell wall to detect the enzymatic activity by zymogram. After electrophoresis, the gel was gently washed in deionized water several times. Then, the gel was transferred into a renaturing buffer, containing 25 mM Tris–HCl, pH 7.2 and 1% Triton X-100, and incubated at 37 °C for 1 h. The gel was stained in a 1% methylene blue solution and washed in deionized water. The results of the zymographic analysis were visualized using the Gel Doc XR+ gel documentation system (Biorad, Hercules, CA, USA).

### 2.6. Antimicrobial Activity Assay

The ability of the endolysin LysAP45 to lyse target bacteria was measured by counting the number of colony-forming units (CFU) after incubation with LysAP45. Each *Staphylococcus* strain was freshly cultured to the exponential phase (OD_600_ = 0.5) and harvested by centrifugation at 4000× *g* for 5 min. The harvested cells were washed and suspended in R-buffer (50 mM Tris-HCl; pH 8.0); the cell suspension was diluted to a titer of 10^6^ CFU/mL, and 100 µL of the cell suspension was placed in each well of a 96-well plate. LysAP45 was diluted in R-buffer or R-buffer containing CaCl_2_, MgSO_4_, ZnCl_2_, or NiSO_4_ at a concentration of 1 μM or 5 μM. The cells were supplemented with LysAP45 in R-buffer or LysAP45 in R-buffer containing CaCl_2_, MgSO_4_, ZnCl_2_, or NiSO_4_ at the concentrations indicated above. The cells that were supplemented with R-buffer or R-buffer containing CaCl_2_, MgSO_4_, ZnCl_2_, or NiSO_4_ at the concentrations of 1 μM or 5 μM were used as controls. The plate was incubated at 37 °C for 1 h; then, aliquots of LysAP45-treated and -non-treated cell suspensions were plated on LB agar plates. The colonies were counted the next day. This experiment was repeated twice, with three replicates.

### 2.7. S. aureus, S. haemolyticus, and S. epedermidis Biofilm Assay

Biofilms were obtained on the surface of coverslips placed in Petri dishes. To prepare the biofilms, *S. aureus* strain CEMTC 1685, *S. epidermidis* strain CEMTC 2043, or *S. haemolyticus* CEMTC 3413 cells were resuspended in 200 µL of a sterile 0.9% NaCl solution to a concentration of 10^9^ CFU/mL. Each bacterial cells suspension was thoroughly mixed with 10 mL of LB and added to a Petri dish containing sterile coverslips. Then, the dishes were incubated for 5 days at 37 °C. On the fifth day, the coverslips with biofilms were removed from the LB medium. The quality of the biofilms was monitored by microscopy (Zeiss Axio Imager A2, Carl Zeiss, Oberkochen, Germany). LysAP45 solutions in PBS (0.1 mg/mL) or in PBS without LysAP45 (negative control) were added to the biofilms, and the samples were incubated for 30 min or 3 h at 37 °C. The biofilms were stained with 0.1% methyl violet, and the results were assessed using a Zeiss Axio Imager A2 microscope (Carl Zeiss, Oberkochen, Germany).

### 2.8. Thermal Stability of LysAP45 Assay

The thermal stability of recombinant LysAP45 was assessed by incubating LysAP45 at various temperatures (50 °C, 60 °C, 70 °C, 80 °C, and 90 °C) for 30 min or 60 min in storage buffer at a concentration of 0.5 mg/mL. The remaining enzymatic activity was evaluated by zymography with *S. aureus* peptidoglycan, as described in Section 2.4.

### 2.9. Statistical Analysis

Statistical analysis was performed using the *t*-test and one-way analysis of variance (ANOVA). The differences between the groups were considered significant at *p* < 0.05. The analysis was carried out using Statistica version 10.0 software (StatSoft. Inc., Tulsa, OK, USA).

## 3. Results

### 3.1. In Silico Characterization of LysAP45

Recombinant LysAP45 contains 288 original aa plus six His aa; it has an estimated molecular weight of 33.7 kDa and an isoelectric point (pI) of 9.8. The solubility of the putative recombinant endolysin LysAP45 was predicted based on two different bioinformatics tools (SoluProt and Protein-sol), and the result showed that the putative recombinant endolysin has sufficient solubility scores (>0.5) when expressed in *E. coli* cells.

The analysis of the LysAP45 aa sequence using BLASTp showed that the similarity with other aa sequences from the Genbank database was less than 56% (*N*-acetylmuramuramoyl-l-alanine amidase from Oceanobacillus neutriphilus, WP_188738042). The InterProScan software package (https://www.ebi.ac.uk/interpro/search/sequence/, accessed on 29 July 2023) was used to map the domain architecture of LysAP45. It was shown that LysAP45 belongs to the superfamily of peptidoglycan recognition proteins (PGRPs) (cd06583) and contains two domains. The N-terminal domain is the *N*-acetylmuramuramoyl-l-alanine amidase-like (NALAA-2, EC 3.5.1.28, EC 3.5.1.28) type II domain (IPR002502) [23,29,30]. In LysAP45 sequence, the NALAA-2-like catalytic domain (pfam01510) is located between the aa residues 23 and 140. Amidases from the NALAA-2 group are zinc-dependent enzymes that hydrolyze the amide linkage between *N*-acetylmuramuramoyl-l-alanine and *L*-amino acids in the bacterial cell wall [23,30]. The C-terminal region of LysAP45 has low similarity with aligned aa sequences from GenBank and was not classified in silico.

**Figure 1:**
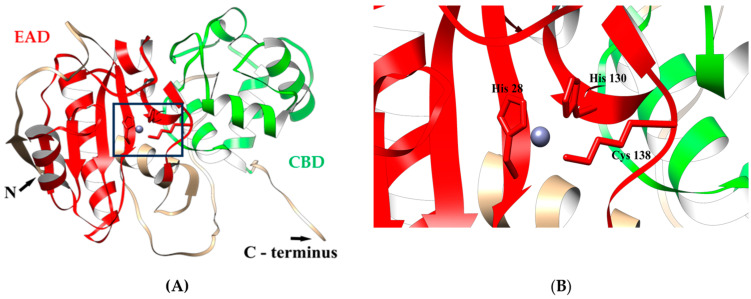
(**A**) Ribbon representation of the predicted 3D structure of endolysin LysAp45 in complex with Zn^2+^ ion; the EAD and CBD are marked in red and green, respectively. (**B**) Ribbon representation of the putative zinc-binding site; His28, His130, and Cys138 are shown in a stick representation. The molecular coordinates of the predicted 3D structure of endolysin LysAp45 were rendered using the UCSF chimera molecular visualizer, version 1.15.

To further investigate the putative structure of LysAP45, a 3D model was predicted using AlphaFold2. It was confirmed that LysAP45 consists of two domains. The N-terminal globular core contains the predicted enzymatic NALAA-2-like domain and connects to the C-terminal globular domain via an unordered linker (Figure 1).

The N-terminal domain contains two α-helices and one β-sheet; the C-terminal domain consists of four α-helices interlinked by flexible linkers and form the cell-binding domain. In addition, the ligand-binding site of LysAP45 was predicted using the meta-server approach to protein–ligand-binding site prediction COFACTOR [28]. It was simulated that the catalytic zinc ion (Zn^2+^) was bound to His28, His130, and Cys138 (Figure 1).

### 3.2. Expression and Purification of LysAP45

**Figure 2:**
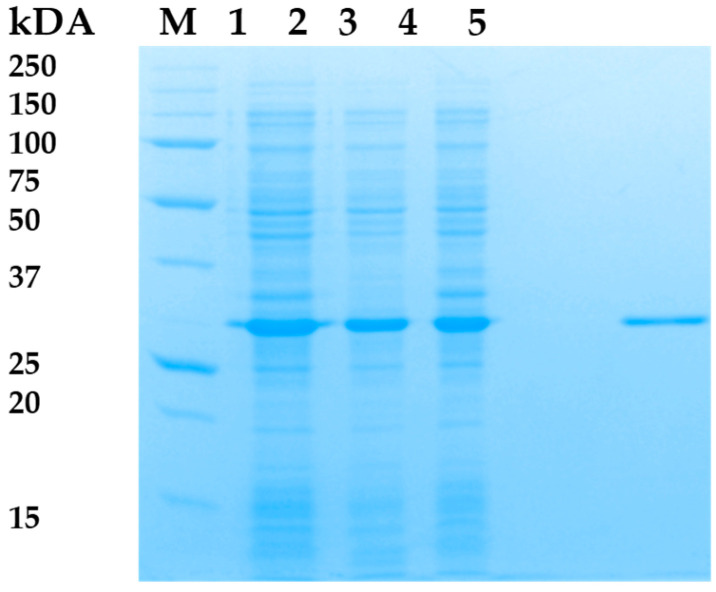
SDS-PAGE. 1—lysates of *E. coli* M15-pQE-70/LysAP45 cells producing recombinant LysAP45; 2—soluble cytoplasm of *E. coli* M15-pQE-70/LysAP45; 3—insoluble cytoplasm of *E. coli* M15-pQE-70/LysAP45; 4—empty well; 5—purified LysAp45 after purification using Ni-NTA chromatography. M-protein ladder Precision Plus Protein™ Standards (Bio-Rad, Hercules, CA, USA).

Attempts were made to optimize the production of recombinant endolysin LysAP45 in order to increase the proportion of soluble LysAP45. Two *E. coli* strains were used, various concentrations of IPTG (10 µM, 100 µM, 300 µM, and 500 µM) were applied, and the transformed cell were cultivated at different temperatures (18 °C, 25 °C, 30 °C, 36 °C). The plasmid pQE-70/LysAP45 was used to transform *E. coli* M15 or *E. coli* DH5alphaF` cells. Protein production was assessed using PAGE. As a result, the level of LysAP45 production after inducing the transformed E. coli M15-pQE-70/LysAP45 cells with 100 µM of IPTG when cultivated at 25 °C was the highest, and ~40% of the produced LysAP45 was in soluble form (Figure 2). Recombinant LysAP45 was purified from the soluble cytoplasmic fraction using metal-chelate chromatography. The evaluated molecular weight of LysAP45 was 33 kDa and corresponded to that predicted in silico (Figure 2). The purity of LysAP45 after chromatography was assessed using PAGE and was ~95% (Figure 2). The yield of LysAP45 production was 20 mg from 1 L of cell culture after purification and dialysis. Purified LysAP45 was stored at a concentration of more than 1 mg/mL in buffer S (50 mM Tris-HCl pH 7.5, 300 mM NaCl) at 4 °C.

### 3.3. Enzymatic Activity of LysAP45

**Figure 3:**
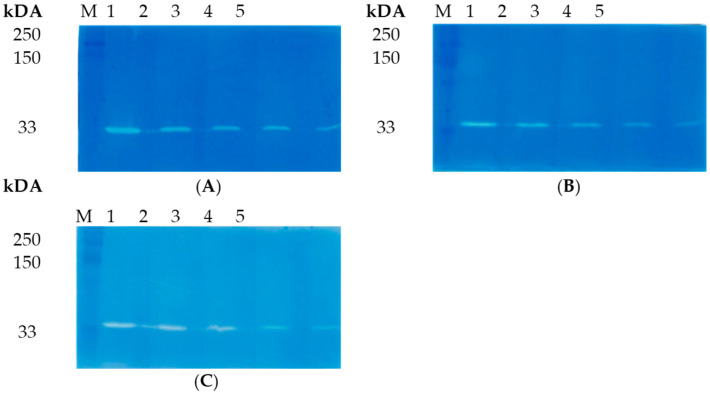
Zymographic analysis of recombinant LysAP45. PAGE (12.5%) containing 0.5 mg of peptidoglycans from *S. aureus* CEMTC 675 (**A**), *S. haemolyticus* CEMTC 3413 (**B**), and *S. epidermidis* CEMTC 2043 (**C**) stained with methylene blue. Lines: 1—LysAP45 (1.6 µg); 2—LysAP45 (0.8 µg); 3—LysAP45 (0.4 µg); 4—LysAP45 (0.2 µg); 4—LysAP45 (0.1 µg); M-protein ladder Precision Plus Protein™ Standards (Bio-Rad, Hercules, CA, USA).

The lytic activity of lysAP45 was evaluated by zymography. Peptidoglycans that were isolated from the cell walls of various *Staphylococcus* strains were used as substrates. It was shown that LysAP45 effectively hydrolyzed peptidoglycan from the cell wall of the *S. aureus* strain CEMTC 1685 (Figure 3). In addition, the lytic activity of LysAP45 was evaluated using peptidoglycans isolated from the cell wall of *S. aureus* CEMTC 675 and *S. aureus* CEMTC 1733, as well as peptidoglycans from coagulase-negative *Staphylococcus* spp., namely, *S. epidermidis* CEMTC 2043, *S. epidermidis* CEMTC 2079, *S. haemolyticus* CEMTC 3413, *S. haemolyticus* CEMTC 3753, *S. warneri* CEMTC 2062, *S. warneri* CEMTC 4154, *S. saprophyticus* CEMTC 3872, and *S. saprophyticus* CEMTC 6829. The results indicated that LysAP45 effectively hydrolyzed peptidoglycans from the cell wall of all the above-listed *Staphylococcus* strains, despite their resistance to antibiotics (Figure 3, Figure S2).

### 3.4. Phage AP45 Host Range

As recombinant LysAP45 showed hydrolytic activity against peptidoglycans from different *Staphylococcus* species, its parental phage AP45 was tested for its ability to infect the above-mentioned *Staphylococcus* strains. All tested strains (*S. aureus* CEMTC 675, *S. aureus* CEMTC 1733, *S. epidermidis* CEMTC 2043, *S. epidermidis* CEMTC 2079, *S. haemolyticus* strain CEMTC 3413, *S. haemolyticus* CEMTC 3753, *S. warneri* CEMTC 2062, *S. warneri* CEMTC 4154, *S. saprophyticus* CEMTC 3872, and *S. saprophyticus* CEMTC 6829) were insensitive to AP45 (Table S1), whereas the host strain *A. pallidus* CEMTC 656 was expectedly susceptible.

### 3.5. Anti-Staphylococcal Activity of LysAP45

**Figure 4:**
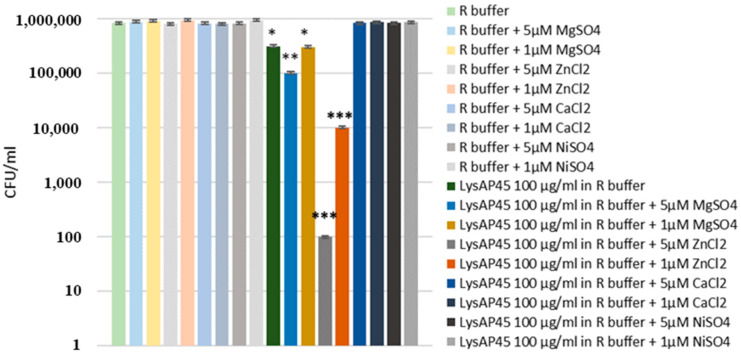
Antibacterial activity of LysAP45 against *S. aureus* CEMTC 1685. LysAP45 (100 µg/mL) was added to cells with or without ions of divalent metals (Zn^2+^, Ni^2+^, Mg^2+^, and Ca^2+^), and the mixtures were incubated for one hour before seeded on LB/agar plates. Cells with only ions of divalent metals was used as a negative control. Experiments were performed in triplicate. *** *p* < 0.001, ** *p* < 0.01, * *p* < 0.05. The *y*-axis is presented on a logarithmic scale.

The antibacterial activity of LysAP45 was tested against a planktonic culture of *S. aureus* CEMTC 1685. It was shown that treatment of *S. aureus* CEMTC 1685 cells with LysAP45 (100 µg/mL) resulted in a 2.7-fold decrease in CFU (8.3 × 10^5^ CFU/mL in the control group, 3.1 × 10^5^ CFU/mL in the LysAP45-treated group) (Figure 4). Then, the effect of different concentrations of CaCl_2_, MgSO_4_, ZnCl_2_, and NiSO_4_ on cell growth was tested. It was found that CaCl_2_, MgSO_4_, ZnCl_2_, or NiSO_4_ insignificantly affect cell growth when used at concentrations ≤5 µM. If LysAP45 was supplemented with CaCl_2_, MgSO_4_, ZnCl_2_, or NiSO_4_ at the concentrations of 1 µM and 5 µM, the results significantly varied. The highest activity was demonstrated by LysAP45 supplemented with ZnCl_2_, and ZnCl_2_ helped to destroy the target bacteria in a dose-dependent manner (Figure 4). The presence of 1 µM ZnCl_2_ in the R-buffer improved the antistaphylococcal efficacy of LysAP45 up to 1000-fold; when 5 µM ZnCl_2_ was added to LysAP45, all *S. aureus* cells died. The addition of MgSO_4_ had a much weaker effect: the presence of 5 µM MgSO_4_ improved LysAP45 activity by 2.7 times, whereas 5 µM MgSO_4_ slightly reduced the effectiveness of LysAP45 (Figure 4). CaCl_2_ and NiSO_4_ inhibited the enzymatic activity of LysAP45.

### 3.6. Thermal Stability of LysAP45

**Figure 5:**
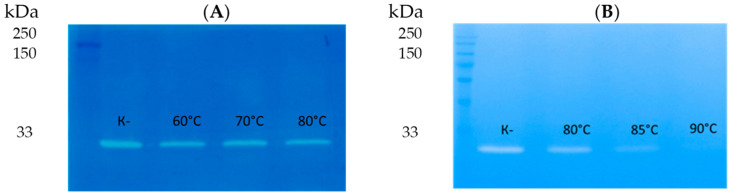
Zymographic analysis of recombinant LysAP45. PAGE (12.5%) containing 0.5 mg of peptidoglycan from *S. aureus* CEMTC 675 after incubation for 30 min (**A**) and 60 min (**B**) at the indicated temperature stained with methylene blue. M-protein ladder Precision Plus Protein™ Standards (Bio-Rad, Hercules, CA, USA).

The effect of high temperature on the enzymatic activity of LysAP45 was evaluated by zymography after incubation of LysAP45 at 50 °C, 60 °C, 70 °C, 80 °C, or 90 °C for 30 min or 60 min. Peptidoglycan isolated from *S. aureus* CEMTC 1685 was used as a substrate. It was revealed that the hydrolytic activity of the recombinant endolysin LysAP45 was not affected after incubation at 50 °C, 60 °C, 70 °C, or 80 °C for 30 min (Figure 5). The enzymatic activity decreased after incubation of LysAP45 at 80 °C for 60 min and disappeared after incubation at 90 °C (Figure 5). Heating at 90 °C for 10 min did not affect the activity of LysAP45.

### 3.7. Biofilm Disruption Activity of LysAP45

**Figure 6:**
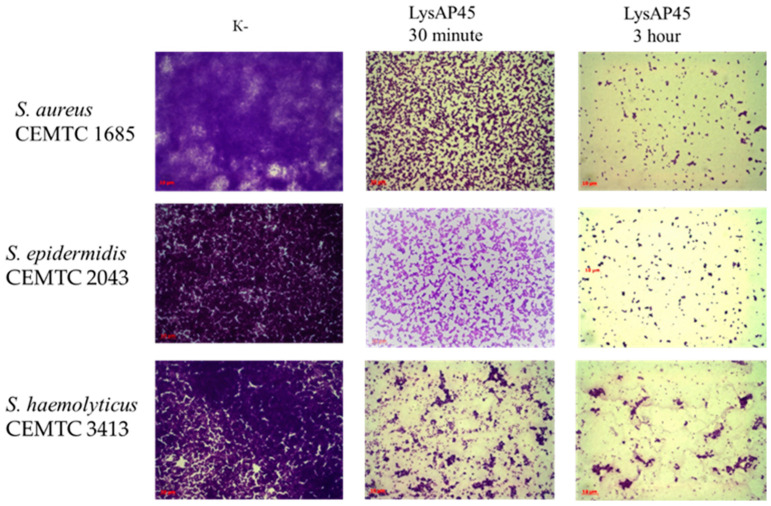
Biofilm disruption activity assay for the recombinant protein LysAP45 against biofilms formed by *S. aureus* CEMTC 1685, *S. epidermidis* CEMTC 2043, and *S. haemolyticus* CEMTC 3413. Bacterial cells were stained with methylene blue. The fields with the maximum and minimum number of cells after incubation with LysAP45 are shown.

The hydrolytic activity of LysAP45 was evaluated using biofilms formed by Staphylococcus strains with multidrug resistance. Strains of different species were selected: *S. aureus* strain CEMTC 1733, *S. epidermidis* strain CEMTC 2043, and *S. haemolyticus* strain CEMTC 3413 (Table S1). Biofilms were prepared on the surface of coverslips placed into Petri dishes. We applied 50 μL of recombinant endolysin LysAP45 at a concentration of 0.1 mg/mL to the grown biofilms, and a sterile storage buffer was added as a control. The biofilms were incubated for three hours at 37 °C with LysAP45. In the control group of biofilms, an extensive matrix was observed, densely filled with bacteria stained with methyl violet (Figure 6). In the biofilms formed by *S. aureus* strain CEMTC 1733, *S. epidermidis* strain CEMTC 2043, and *S. haemolyticus* strain CEMTC 3413 treated with LysAP45, only remnants of a biofilm matrix were observed (Figure 6). Only a small number of staphylococcal cells was still found in the biofilm matrix compared to that observed in the control samples (Figure 6).

## 4. Discussion

Endolysins for the therapy of bacterial infections are a promising platform for the development of new antimicrobial drugs capable of treating staphylococcal infections with a low risk of inducing drug resistance. Usually, recombinant analogues of endolysins as new therapeutic agents were obtained from mesophilic phages [31]. Such endolysins often have high lytic activity [16,18,19,32,33]; however, they are inactivated at high temperature or low pH, which is a disadvantage in biotechnological processes [31]. Considering these possibilities, the novel thermostable endolysin LysAP45 was characterized in this study.

The gene encoding LysAP45 was identified from the genome of phage AP45 isolated from a sample collected in the Geyser Valley, Kamchatka Peninsula [21]. The AP45 genome was shown to be unique and significantly different from the genomes of other known mesophilic and thermophilic phages [21]. The AP45 phage showed a narrow host range, being specific to two *A. pallidus* strains. This phage did not infect 25 *A. pallidus* strains and other thermophilic bacterial strains collected in the Geyser Valley and Baikal Rift Zone [21]. The recombinant endolysin LysAP45 was developed using the AP45 genome, and the ability of LysAP45 to hydrolyze peptidoglycans from the host strain *A. pallidus* CEMTC 656 was shown [21].

In this study, the conditions for LysAP45 production were optimized, and a high yield of the target endolysin was achieved. Unexpectedly, we found that LysAP45 was able to hydrolyze peptidoglycans from various Staphylococcus spp., both coagulase-positive and coagulase-negative. Notably, the parental AP45 phage does not infect the *Staphylococcus* spp. strains used in this study. Given the unexpected specificity of LysAP45, its structure was analyzed in silico. Usually, endolysins of phages of Gram-positive bacteria have a well-defined domain structure and contain two types of domains—the EAD, more often located at the N-terminus of endolysin, and a C-terminal CBD [9]. The topology of LysAP45 was ordinary. The N-terminal domain was predicted as an amidase from the NALAA-2 group. The ligand-binding site of NALAA-2-like amidases often contains the catalytic Zn^2+^ ion [17]. We tested the lytic activity of LysAP45 in the presence of Zn^2+^, Ni^2+^, Mg^2+^, and Ca^2+^. Among these ions, Zn^2+^ increased the lytic activity of LysAP45 by 1000–100,000 times depending on its concentration, which confirmed that the enzyme is a zinc-dependent amidase. In LysAP45, Zn2+ presumably binds to His28, His130, and Cys138 (Figure 1). We assume that the various effects of divalent ions on LysAP45 activity are due to the structure of the Zn-binding site. Probably, His28, His130, and Cys138 form such a coordination site that only Zn^2+^ ions can bind to it with high selectivity and affinity, while other divalent ions either do not bind or interact with low affinity. For instance, Zn-binding sites in metalloproteins are highly specific for Zn^2+^ ions and bind other divalent ions more poorly [34].

Importantly, the C-terminal CBD of LysAP45 was unique and showed no similarity to any CBD sequences from other described endolysin sequences extracted from GenBank. For further investigation of the putative CBD, two variants of the recombinant protein were developed: CBD (C-terminal part of LysAP45, 163–288 aa) and CBD fused with the N-terminal thioredoxin (Trx) to improve solubility. Unfortunately, our attempts to obtain the active CBD domain were unsuccessful. Both CBD and Trx-CBD were completely insoluble after production in different *E. coli* strains using various cultivation conditions. After refolding from inclusion bodies and subsequent dialysis, inactive CBD and Trx-CBD precipitated. So, the main limitation of this study is the inability to investigate the specificity of this unusual CBD. It should be noted that the sequence of the C-terminal domain of LysAP45 is unique, and we can only assume the localization of the CBD in this sequence region. Further investigations are required to determine the specificity of LysAP45 and its putative CBD in Gram-positive bacteria.

The bacterial host of AP45, A. pallidus CEMTC 656, was isolated from a Geyser Valley soil sample, and the optimal growth temperature for this strain is from 50 °C to 65 °C [21]. Expectedly, the phage AP45 was more thermostable than its host and survived when incubated at 85 °C for 24 h and at 95 °C for 1 h. The thermal stability of LysAP45 was lower than that of AP45 (70–80 °C), since this protein is produced by cells that prefer to live at a temperature of 55 °C.

Several thermostable phage endolysins from both mesophilic and thermophilic bacteria have been previously described [35,36,37,38,39,40,41,42,43,44,45]. Thermostable endolysins derived from thermostable bacteriophages are usually most active at temperatures above the physiological values. Previously, the antistaphylococcal activity of thermostable endolysins was described in several studies. Thus, the endolysin TP84_28 of the Geobacillus stearothermophilus phage TP-84 showed the ability to destroy biofilms formed by *S. aureus* after incubation for 24 h at 37 °C with further incubation for 1 h at 55 °C; however, the lytic activity of the endolysin towards planktonic *S. aureus* cultures was not described [42]. The lytic activity against *S. aureus* planktonic cultures was shown for the thermostable TSPphg lysine from the Thermus phage TSP4 and the peptidoglycan hydrolase HydH5 of the *Staphylococcus* phage vB_SauS-phiIPLA88; however, the ability to destroy biofilms formed by staphylococci has not been described for both enzymes [43,44]. The thermostable endolysin P28 of the Stenotrophomonas maltophilia phage exhibited weak lytic activity against *S. aureus* at high concentrations (0.5 mg/mL); however, there is no information about its ability to destroy biofilms [45]. This study described the first thermostable phage endolysin that is able to hydrolyze *S. aureus* planktonic cultures and disrupt biofilms formed both coagulase-positive and coagulase-negative staphylococci. The stability of LysAP45, even at 70–80 °C, may be useful for the biotechnological production and storage of this endolysin.

## 5. Conclusions

In this study, the production of the recombinant LysAP45 endolysin from the *A. pallidus* AP45 phage was improved. Despite this phage having a narrow host range and infecting only *A. pallidus* (the Caryophanales order), LysAP45 was able to hydrolyze peptidoglycans from both coagulase-positive and coagulase-negative *Staphylococcus* spp. (Bacillales order). Thermostability tests showed that LysAP45 had enzymatic activity after incubation at 80 °C for at least 30 min. In silico analysis indicated that LysAP45 consists of two domains: the EAD belonging to the NALAA-2 type and a putative CBD that has no similarity with the protein sequences from the database. It was revealed that LysAP45 eliminated the biofilms formed by multidrug-resistant *S. aureus*, *S. epidermidis*, and *S. haemolyticus* in 30 min. The results suggest that LysAP45 is a novel thermostable antimicrobial agent capable of disrupting biofilms formed by various species of staphylococci, including MDR strains.

## Data Availability

The data presented in this study are available on request from the corresponding author.

## Supplementary Materials

The following supporting information can be downloaded at https://www.mdpi.com/article/10.3390/v16010093/s1, Figure S1: Map of the plasmid pQE-70/LysAP45. Figure S2: Zymographic analysis of recombinant LysAP45. PAGE (12.5%) containing 0.5 mg of peptidoglycan from *S. aureus* CEMTC 1733 (A), *S. epidermidis* CEMTC 2079 (B), *S. haemolyticus* CEMTC 3753 (C), *S. warneri* CEMTC 2062 (D), *S. warneri* CEMTC 4154 (E), *S. saprophyticus* CEMTC 3872 (F), and *S. saprophyticus* CEMTC 6829 (G) stained with methylene blue. Table S1: Sensitivity of *Staphylococcus* strains to antibiotics and the AP45 phage.
