# Supplementary material for: Characterization of a Thermostable Endolysin of the Aeribacillus Phage AeriP45 as a Potential Staphylococcus Biofilm-Removing Agent
Source: Viruses. 2024 Jan 7;16(1):93. doi: 10.3390/v16010093 (PMC10819204; doi:10.3390/v16010093)
Supplement: Supplementary file 1 [file viruses-16-00093-s001.zip › Figure S1.pdf]

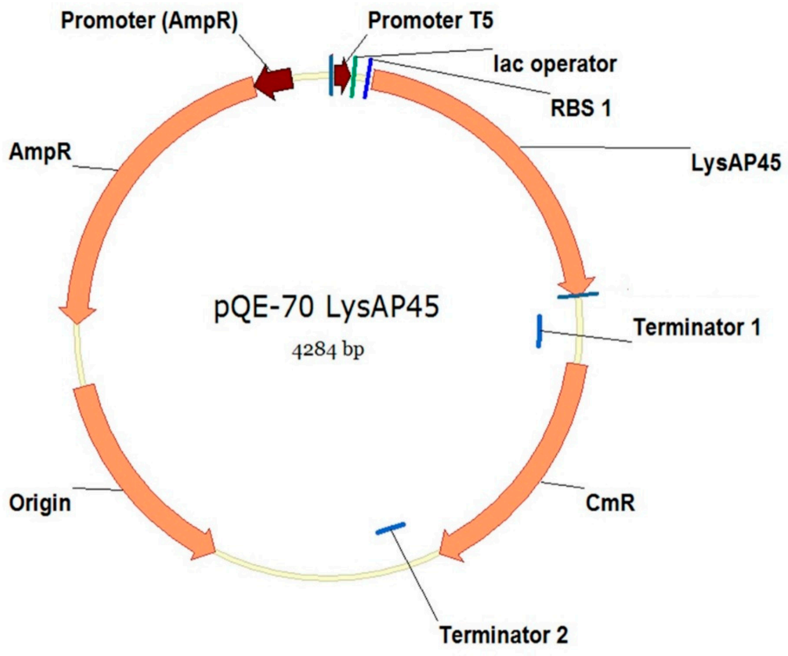

Figure S1. Map of the plasmid pQE-70/LysAP45. RBS-ribosome binding site. LysAP45 – gene encoded endolysin LysAP45. AmpR – ampicillin resistance gene.
