# Supplementary material for: Characterization of a Thermostable Endolysin of the Aeribacillus Phage AeriP45 as a Potential Staphylococcus Biofilm-Removing Agent
Source: Viruses. 2024 Jan 7;16(1):93. doi: 10.3390/v16010093 (PMC10819204; doi:10.3390/v16010093)
Supplement: Supplementary file 1 [file viruses-16-00093-s001.zip › Figure S2.pdf]

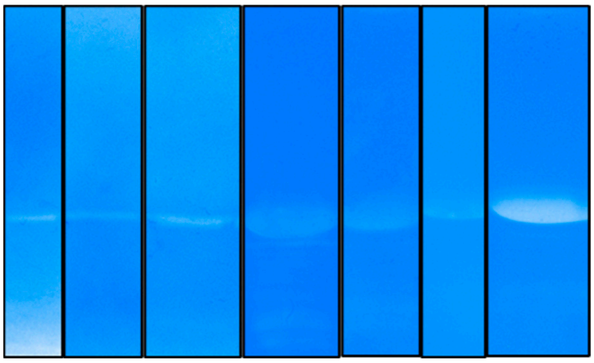

Figure S2. Zymographic analysis of recombinant LysAP45. PAGE (12,5%) containing 0.5 mg of peptidoglycan from *S. aureus* CEMTC 1733 (A), *S. epidermidis* CEMTC 2079 (B), *S. haemolyticus* CEMTC 3753 (C), *S. warneri* CEMTC 2062 (D), *S. warneri* CEMTC 4154 (E), *S. saprophyticus* CEMTC 3872 (F), and *S. saprophyticus* CEMTC 6829 (G) stained with methylene blue.
