# Supplementary material for: Characterization of a Thermostable Endolysin of the Aeribacillus Phage AeriP45 as a Potential Staphylococcus Biofilm-Removing Agent
Source: Viruses. 2024 Jan 7;16(1):93. doi: 10.3390/v16010093 (PMC10819204; doi:10.3390/v16010093)
Supplement: Supplementary file 1 [file viruses-16-00093-s001.zip › Table S1.pdf]

Table S1. Sensitivity of *Staphylococcus* strains to antibiotics and the AP45 phage

Resistance to Antibiotics											
**Antibiotic disc**	*S. aureus* CEMTC			*S. epidermidis* CEMTC		*S. haemolyticus* CEMTC		*S. warneri* CEMTC		*S. saprophyticus* CEMTC	
	675	1685	1733	2043	2079	3413	3753	2062	4154	3872	6829
**β-lactams**	FOX	s	P, OX/FOX	CFM	FOX	FOX	FOX	s	P	s	P
**Aminoglycosides**	CN	s	CN	s	s	AK, CN	s	s	AK, CN	s	s
**Macrolides**	E,	s	E	E	E	E	E	E	s	s	s
**Lincosamides**	DA	LNM	s	s	s	s	s	s	s	s	s
**Fluoroquinolones**	s	s	CIP, LEV,	CIP, LEV,	s	s	s	s	s	s	s
**Chloramphenicol**	s	s	C	C	s	s	s	s	s	s	s
**Tetracyclines**	s	s	s	s	s	s	s	s	s	s	s
**Glycopeptides**	s	VA	s	VA	s	s	s	s	s	s	s
**Oxazolidinones**	s	s	s	s	s	s	s	s	s	s	s
	MDR	R	MDR	MDR	R	MDR	R	R	R	S	R
**Sensitivity to the AP45 phage**											
**AP45 phage**	i	i	i	i	i	i	i	i	i	i	i

FOX – Cefoxitin; P – Penicillin; OX – Oxacillin; CFM – Cefixime; AK – Amikacin; CN – Gentamicin; E – Erytromycin; DA – Clindamycin; LNM – Lincomycin; CIP - Ciprofloxacin, LEV – Levofloxacin; C - Chloramphenicol; VA – Vancomycin. S – sensitive, i - insensitive
